# Dropout of infertility treatments and related factors among infertile couples

**DOI:** 10.1186/s12978-020-01048-w

**Published:** 2020-12-02

**Authors:** Maryam Ghorbani, Fatemeh Sadat Hosseini, Masud Yunesian, Afsaneh Keramat

**Affiliations:** 1 Student Research Committee, School of Nursing and Midwifery, Shahroud University of Medical Sciences, Shahroud, Iran; 2 Faculty Member of Medical School, Golestan University of Medical Sciences, Gorgan, Iran; 3 School of Public Health, Tehran University of Medical Sciences, Tehran, Iran; 4 Reproductive Studies and Women’s Health Research Center, Shahroud University of Medical Sciences, Shahroud, Iran

**Keywords:** Study protocol, Sequential exploratory mixed-method study, Infertility, Treatment dropout

## Abstract

**Background:**

Dropout of infertility treatments is a global issue and many factors play role in this phenomenon. It is one of the most challenges in life of infertile couples. The purpose of this study was to determine dropout rate and related factors/reasons in the world and in Iran.

**Methods:**

We will conduct a mixed method study with sequential exploratory design (systematic review, qualitative and quantitative phase). In the first stage a systematic review on dropout rate of infertility treatments and related factors will be done. In second stage (quantitative–qualitative study), a retrospective cohort study will be conducted on infertile couples to determine dropout rate of infertility treatments. The follow-up period to assess the discontinuation of treatment in patients, who have discontinued the treatment, will be considered 6 months after the treatment cessation. Data would be analyzed by descriptive statistics. We want to determine proportion and percentage of discontinuation rate among different groups with different causes of infertility. Then, we also will use Chi-square test to compare discontinuation rates among these groups. In qualitative section of second stage, semi-structured interviews would be performed with infertile female who had the history of infertility treatments failure. In this stage, participants will be selected using purposeful sampling method with maximum variation in terms of age, education, occupation, type of infertility, type of treatments, number of unsuccessful treatment and infertility duration. Data would be analyzed using conventional content analysis.

**Discussion:**

Determining dropout rate and its related factors/reasons would be helpful for future studies to plan suitable interventions for supporting infertile couples. It also helps politicians to have a better understanding of infertility and its consequences on infertile couple’s life.

**Plain English Summary:**

In today’s world, infertility is a common phenomenon due to postponement of childbearing following the older age of marriage, tendency to reach higher educational level, economical problems and etc. Infertility brings many challenges and stresses to the individuals by itself and it is very hard to cope with. The problem gets worse, when it is associated with failure in treatments. Many of infertile couples cannot tolerate this failure and may decide to discontinue treatments before achieving pregnancy for ending many stressors which are associated with treatments. As we know, childbearing and having at least one child has important position in some societies such as Iranian culture; so ending the treatment before achieving optimal result may have some adverse consequences in the families such as divorce, remarriage, family conflicts, et. Absolutely many factors play role in dropout of infertility treatments, and many studies around the world have suggested many factors/reasons in dropout of infertility treatments, but there are still many gaps about this subject, especially among Iranian society. This study would be conducted in three consecutive stages, in the first stage; we will do a complete review of existing studies of the world to find out related factors/reasons of dropout in detail. In second stage, dropout rate of infertile couples (380 couples) after at least one unsuccessful cycle of treatment would be achieved by assessing medical records and telephone interview. Data of the first and second stage will help us to have better vision about the issue of dropout and would be used to construct a semi structured interview for the last stage. And finally in the third stage, reasons of dropout would be asked by an in depth interview from infertile couples. We hope the information from this study will help politicians better understand and plan for dropout of treatment.

## Introduction

Infertility is a clinical condition which means no clinical pregnancy after 12 months of unprotected and regular sex, resulting from a defect in a person's ability to reproduce as an individual or with a male/female partner [1]. It affects 13.2% of couples in Iran [2], and 60–80 million couples of reproductive age in the world [3]. Among the infertile couples, about 56% seek medical help for getting pregnant [4]. The Assisted Reproductive Technology (ART) is the assistance that usually applied to describe medical techniques that increase the probability of pregnancy. The techniques include the In Vitro Fertilization (IVF), Stimulation of Ovulation, and methods that lead to the manipulation of eggs or sperm or involvement of donated eggs and sperm in the laboratory [5, 6].

The success rates of assisted reproductive technologies are relatively constant with about 25% of live births per cycle occurring by the age of 35, after which it declines sharply. This rate of success seems desirable, but it also means that the failure rate is about 75% that is distressing for people who bear heavy financial and psychological costs of these treatments [7]. Therefore, many couples do not continue treatment until a reasonable result is achieved.

The dropout of fertility treatments refers to the further treatment despite the favorable progress and the ability to pay for treatment that can occur at any stage of the treatment [8]. The prevalence of discontinuation varies from 5.6% to 70% in different studies [9–15]. In Iran, the rate of discontinuation of treatment was 56.5% in one study [16] and 28.3% in another study [17]. The variety in prevalence is due to the use of different concepts and definitions [18]. Research on discontinuation of treatment began in 1980 and was motivated by the need to understand its impact on the effectiveness of fertility treatments as well as the reason why some couples did not continue the treatment [4]. Several studies have sought to understand why patients do not continue the treatment. According to the studies, the psychological burden was the most common reason cited by the patients for not continuing the treatment, and there were relationships between not continuing the treatment and the physical burden of treatment, feelings of futility associated with treatment, marital and personal problems, poorly understood diagnosis, parity, and demographic-social factors [4, 8, 18–21].

Due to the heterogeneity of costs, access to infertility treatment services, reimbursement policies, etc., it is difficult to compare the treatment withdrawal rates between centers and countries. In addition, most fertility experts are willing to report positive outcomes and success rates, and tend to ignore or forget the "invisible patients" who discontinue the treatment. Due to the lack of sufficient study on the rate of discontinuation of treatment and its associated factors in Iran, the present study aimed to investigate the dropout rate of treatment and its relevant factors.

## Main objective

- Determining the continuation/dropout rate of treatments and factors affecting the continuation of treatment in infertile couples.

## Specific objectives

### Objectives of the first stage (systematic review)

- Identifying the factors affecting the continuity/dropout of infertility treatments in studies worldwide

## Objectives of the second stage (conducting a quantitative–qualitative study to determine the acceptance of infertility treatments and its determinants)

### Quantitative study objectives

- Determining the continuation/dropout rate of treatment in infertile couples who visit the certain infertility treatment centers.
- Determining the continuation/dropout of treatment according to the cause of infertility (male, female, unexplained, both) in infertile couples who visit the certain infertility treatment centers.
- Determining the continuation/dropout of treatment according to the type of infertility treatment in infertile couples who visit the certain infertility treatment centers.

## Objectives of qualitative study

- Explaining the concept of treatment continuation/dropout and its determinants in infertile couples
- Explaining the dimensions of treatment continuation/dropout and its determinants in infertile couples

## Methods/design

This is a sequential exploratory mixed-method study, with multi stages; the systematic review and quantitative–qualitative studies.

## Stage 1: systematic review

### Search strategy

We will do a comprehensive search in international databases including; Medline (via PubMed), Embase, Web of Science, Cinhal, Proquest and Scopus to find the relevant published papers to the subject of the study from the beginning of 2000 to September 2019. A systematic search will be done by using Mesh terms including “fertility treatment”, “artificial insemination”, “assisted reproductive technology”, “in vitro fertilization”, “intra cytoplasmic sperm injection”, “intra uterine insemination”, ‘IUI”, “ICSI”, “IVF”, AND “discontinuation” OR “dropout” OR “cessation” OR “end” OR “stop” OR “end” OR “termination” OR “withdraw” OR “abandon”. To find relevant papers, we will also perform manual search in Google scholar and checked references of retrieved articles.

## Stage 2: Quantitative–qualitative study to determine the continuation of treatment and explain the factors affecting the continuation of treatment

At the quantitative stage, a retrospective cohort study will be conducted to determine the rates of infertility treatment in women who are referred to the infertility treatment centers for the infertility treatment.

### Research population

The statistical population consists of 18–42 year-old women of reproductive age who are considered infertile according to the global definition of infertility, and visit the infertility treatment centers to receive the IVF/ICSI (Intra Cytoplasmic Sperm Injection), and FET (Frozen Embryo Transfer) services.

### Research sample

The research samples are 420 infertile women (by considering 10% as attrition of samples) (105 women in each of four subgroups based on the cause of infertility: male, female, unexplained, and both).

We measure the sample size based on the following equation:

$$n=\frac{{Z}_{1-\frac{\alpha }{2}}2*P(1-P)}{{d}^{2}}$$

where, α = 0.05 →${Z}_{1-\frac{\alpha }{2}}$ = 1.96: is the Percentile of a normal distribution of the Type I error (5%).

P = 0.56: refers to the estimation of the prevalence of discontinuation of infertility treatments in studies [16].

d^2^ = 0.1 (research precision).

### Characteristics of research samples

- Women at the childbearing age of 18–42 years who are considered infertile according to the global definition of infertility.
- To have at least an unsuccessful IVF/ICSI and FET cycle
- Having a file in the infertility center
- Voluntary participation and written consent
- The Iranian nationality (couple)
- No addiction to narcotics and psychotropic drugs
- Fluency in Persian

### Exclusion criteria

Unwillingness to continue cooperating at any stage of the study and getting pregnant.

### Sampling method

We perform a simple random sampling due to descriptive nature of this stage. In this case, a semi-private facility and a Charity-public center are selected for sampling to increase diversity in the participants. Due to the fact that the patients' visit of these centers do not have a specific time pattern, patients who visit consecutively, can be considered as a random sample of patients. Accordingly, a questionnaire of demographic factors (including questions about age, marital status, age of marriage, age of first menstruation, number of pregnancies, number of deliveries, number of children, number of abortions, duration of infertility, number of infertility treatments, education level, socio-economic status, employment status, and type of client's job, employment status, and type of spouse's job, spouse's education level, ethnicity, insurance status, place of residence) will be completed through the interviews, and the patients' file information will be used to ensure the accuracy of data during a year (2018) and in a retrospective cohort study of all couples who are infertile according to the definition, met the inclusion criteria, and visited the infertility treatment centers. Therefore, the continuation of treatment and non-continuation of infertility treatments will be calculated. It should be noted that the follow-up period to assess the discontinuation of treatment in patients, who have discontinue the treatment, will be considered 6 months after the treatment cessation according to research by Moini et al. [16].

### Data analysis method

Descriptive statistics including frequency, mean and standard deviation tables will be used to express the characteristics of the research units. For example:

a We will use mean and standard deviation to describe age of female and male participants, duration of marriage, marriage age, duration of infertility and menarche age. We also will use ANOVA test to compare theses variables among groups with different causes of infertility.
b Frequency and proportion will be used for theses variables: educational level of female and male participants and income level of family. We will use Kruskal Wallis test to compare theses variables among groups with different causes of infertility.
c We will use frequency and proportion to describe ethnicity of male and female participants, residential place, number of pregnancy, number of abortion and number of live child. Chi square test will help us to compare theses variables among groups with different cause of infertility.
d To compare relationship between different variables such as age groups, educational levels of male and female participants, ethnicity of male and female participants, income level of family, age groups, cause of infertility and continuation or dropout of treatment, we will use Chi-square test.

We want to determine proportion and percentage of discontinuation rate among different groups with different causes of infertility. Then, we also will use Chi-square test to compare discontinuation rates among these groups. Confidence interval of 95% and significance level of α = 0.05 will be considered in all tests. SPSS 21 will be used to analyze data.

At the qualitative stage of study, since the effective factors in discontinuing the infertility treatments are complex and different and not well known in the Iranian society, a qualitative study with content analysis and a conventional approach will be conducted to achieve the second stage objective, namely "determining the acceptance of infertility treatments and its determinants".

### Data collection

Data will be collected through in-depth semi-structured interviews with infertile women who are eligible for participating in the study at least after 6 months of the last unsuccessful IVF/ICSI or FET. Participants will be selected using purposeful sampling method with maximum variation in terms of age, education, occupation and infertility duration, type of infertility (primary of secondary), type of infertility treatment and number of unsuccessful treatments. Purposive sampling technique is a technique in which researcher relies on his or her own judgment when choosing members of population to participate in the study. Therefore, now, it is not possible for us to determine sample size (and number of participants based on their characteristics) and we must wait the result of previous stages to choose our participants with purpose. Also, by selecting participants from both semi-private and public (charitable) centers, efforts will be made to provide the maximum possible diversity for the researcher. In qualitative studies, sample size is unpredictable; therefore, we cannot specify sample size and must keep on interviews until reaching saturation, which is commonly used in qualitative research. Saturation provides an indication of data validity [22]. While collecting the data, the interviews will be analyzed using a conventional qualitative content analysis method.

In addition, before starting interview, a General Health Questionnaire (GHQ-28) will be completed to assess the mental health status of the participants [23].

### Participants' characteristics

Research participants will include infertile women who have been diagnosed infertile according to the definition of infertility, with at least a history of unsuccessful IVF/ ICSI or FET treatment, and willingness to participate in the research. They also have the ability to communicate effectively and transfer their experiences in the field of research.

### Research environment

The interviews are individually conducted in quiet places, including the clinics of Jihad Daneshgahi Infertility Center and Nekouei Hospital of Qom. The place and time of interviews will be determined based on the interviewees' desire, preference and convenience. Prior to the interviews, the informed written consent will be obtained from the participants. The interviews will be individually conducted in a quiet place. The approximate time of each session will be announced in advance for the participants and will increase or decrease according to their desire.

### Data analysis method

The content analysis will be performed with a conventional approach along with data collection using a method proposed by Graneheim and Lundman, including the following steps:

1. Implementing the entire interview immediately after each interview.
2. Reading the whole text for a general understanding of its content.
3. Determining the meaning units and basic codes.
4. Classifying similar primary codes into more comprehensive classes.
5. Determining the main themes of classes [24].

### Accuracy and stability of data

We will use four criteria presented by Lincoln and Guba, including the Credibility, Confirmability, Dependability, and Transferability to evaluate the accuracy and reliability of data [25]. To this end, we determine the credibility using the peer and faculty member's check to approve and modify the codes and classifications. Therefore, the supervisors and advisors will review and examine the data obtained from the interviews after implementation and coding. After the data analysis, two participants will be contacted and they will receive a full text of the interview coding to determine their relevance to the participants' experiences. To determine the confirmability, two reproductive health faculty experts will be then asked to study the interviews, codes, and themes. In order to increase the dependability, two experts in the quality work will be asked to re-encode the coding process to identify any inconsistencies in coding. Also, the researcher will encode the interviews again two weeks after the initial coding (code-barcode); and its research steps and methodology, coding process, and decisions at different stages will be described in details so that it will make possible for others to pursue research if necessary. Also, all documents and evidence will be safely stored. To determine the transferability, two participants with inclusion criteria, but not a part of the research community, will receive the coding results and their opinions will be recorded. A comprehensive description of the type of research, the participants' characteristics, and their experiences will be provided for readers in the final report of the study.

## Discussion

Dropout of infertility treatments is a common phenomenon in IVF (In Vitro Fertilization)/ICSI (Intra cytoplasmic Sperm Injection) procedures and many patients avoid continuing infertility treatments [26–32]. In literature, many factors have been suggested for couple’s decision to stop further treatment, such as psychological and physical burden, financial reasons and poor prognosis [12, 19, 33, 34]. But if we had a quick look to the studies reporting the reasons/factors of dropout show that, almost all of them, had the quantitative method for assessing the reasons. But it seems that we need qualitative method with in depth interview to gain worth information about this phenomenon. On the other hand, knowledge about rate and related factors of dropout in Iranian society is limited and there are many gaps for this issue. To our knowledge, this is the first multi methods q study, probing the rate and reasons of dropout in several stages especially with in-depth interview. The result of this study will help politicians to know better the reasons of dropout and have better programs to encourage infertile couples to continue their treatments. The other strong point of the present study is using selecting a large number of participants from infertile women with variety of characteristics. It is possible that in the present study, some of the participants would not express all of truth about infertility, treatments and reasons of dropout which would be one of the limitations of this study. However, we would make efforts to gain the trust of the participants and establishing a relationship with them for resolving this limitation.

## Data Availability

Not applicable.

## Abbreviations

ART: Assisted Reproductive Technology
IVF: In Vitro Fertilization
ICSI: Intra Cytoplasmic Sperm Injection
FET: Frozen Embryo Transfer
GHQ-28: General Health Questionnaire
